# Regulating local environments towards enhanced electrochemical performance

**DOI:** 10.1093/nsr/nwae460

**Published:** 2024-12-16

**Authors:** Chong Liu, Xiangfeng Duan

**Affiliations:** Department of Chemistry and Biochemistry, University of California, Los Angeles, USA; Department of Chemistry and Biochemistry, University of California, Los Angeles, USA

Electrochemistry has long been a cornerstone of modern science, underpinning advancements in energy storage, catalytic transformation and materials science. Typically occurring at the buried interface between solid electrodes and liquid electrolytes, local microenvironments at electrochemical interfaces are inherently different from those of bulk electrolytes. These microenvironments, defined collectively by many factors, including specific surface adsorbates, local solvation structures, ionic distributions and interfacial electric fields within the double layer, exert a profound influence on electrochemical kinetics, reaction pathways and the ultimate reactivity and selectivity in electrochemical transformation [1]. Moreover, the significance of local environments extends beyond fundamental studies; they offer a versatile degree of freedom for tailoring and optimizing the specific electrochemical processes crucial for addressing challenges in climate change, environmental remediation and sustainable chemical manufacturing. Recent advances have offered unprecedented insights into these dynamic interfaces, offering pathways to design and optimize electrochemical processes. In such a context, one cannot help but ask: what's next?

With this question in mind, this special topic of *National Science Review* aims to bring together perspectives, reviews and research articles that explore the nuances and excitements of local environments in electrochemistry. It is proposed that the special topic will underscore the critical role of tailored microenvironments in pushing the frontiers of electrochemical research. It is fascinating that the inquiry into electrochemical local environments is so multifaceted, thanks to its multidisciplinary and complex nature; researchers from various backgrounds have different perspectives on this interesting scientific topic. Among the four perspectives included in this special topic, Qiao *et al.* explored how the choice of solvents and cation-specific interactions will yield encouraging strategies to fine-tune reaction selectivity [2]; Kim *et al.* delved into the interplay between interfacial electric fields and ion interactions, emphasizing their role in modulating catalysis [3]; Schreier *et al.* focused on the entropic contributions within the electrochemical double layers as well as their implications for catalytic activities [4]; Zhang *et al.* discussed the intricate dynamics of interfacial water molecules and their effect on electrocatalytic efficiency, and highlighted molecule-level interactions at electrochemical interfaces [5]. Together, those converging yet differentiating perspectives not only elucidate the fundamental questions of local environments in electrochemistry at different length scales, but also promise the reactivity control that is possible from electrochemistry.

The two research articles in this special topic resonate well with the challenges and promises of local environments in electrochemistry. Li *et al.* reported that the design of PdCu alloys with different geometries (nanoparticles and nanodendrites) can lead to distinct local strain profiles to modify CO_2_ reduction reaction pathways, underscoring how the nanoscopic geometries influence the local environment and reactivity in electrochemistry [6]. Duan *et al.* reported that an elaborate encapsulation of copper nanowires in graphdiyne functionalized with specific electron-withdrawing and electron-donating groups can distinctly modify the nanowire surface electronic structure, thereby favoring different CO_2_ reduction products [7]. This laudable example illustrates that fine-tuning the local electronic structure of the surfaces of electrocatalysts promises a tailored electrocatalytic performance.

So, what's next in the seemingly never-ending inquiry into local environments in electrochemistry? As we continue unraveling the complexities and beauty of electrochemical interfaces, our hope is that the work presented here will inspire new lines of inquiry and foster innovation across disciplines. As a concluding remark and a stepping stone for future research, the review of Chen *et al.* presents the advances in microenvironment regulation strategies in electrocatalysis [8]. This timely review summarizes a wide range of electrochemical applications ranging from water splitting to CO_2_ electrocatalysis, which offers a useful guidebook for next-generation researchers. By deepening our understanding of local environments in electrochemistry, we are moving closer to addressing the pressing challenges of our time, such as climate change, with a powerful electrochemical toolbox.

Last but not least, the completion of this special topic owes much to the collaborative efforts of many individuals. We extend our deepest gratitude to the authors for their insightful contributions, the reviewers for their meticulous evaluations, and the editorial staff of *National Science Review* for their unwavering support.
